# Morphology and sexual dimorphism of living mature adults of *Amphiduros fuscescens* (Marenzeller, 1875) (Annelida, Hesionidae, Amphidurine), first reported for the Iberian Peninsula

**DOI:** 10.3897/BDJ.9.e66020

**Published:** 2021-05-19

**Authors:** Daniel Martin, Chiara Romano

**Affiliations:** 1 Centre d'Estudis Avancats de Blanes (CEAB - CSIC), Blanes, Spain Centre d'Estudis Avancats de Blanes (CEAB - CSIC) Blanes Spain

**Keywords:** Polychaete, Hesionid, sexual dimorphism, biogeographic distribution, Mediterranean Sea, COI

## Abstract

**Background:**

To date, the genus *Amphiduros* (Annelida: Hesionidae: Amphidurine) is considered as monotypic. Its single species, *Amphiduros fuscescens* (Marenzeller, 1875), is well characterised by lacking proboscideal papillae and emerging acicular chaetae, as well as by having three antennae, eight pairs of tentacular cirri and inflated dorsal cirri with characteristic alternating length and colour (transparent, with median orange band and white tips) in live animals.

**New information:**

Three specimens, one male and two females, were found below boulders at 5–7 m depth in Punta Santa Anna, Blanes and Cala Maset, Sant Feliu de Guixols (Catalan Sea, NW Mediterranean, Iberian Peninsula). Our finding allowed us to describe different, unreported morphological traits and lead us to support the existence of sexual dimorphism (in terms of colouring, cirri morphology and distribution of sexual products along the body). Despite *A. fuscescens* having been previously reported from the Atlantic and the Mediterranean (particularly in SE French coasts), the specimens from Blanes represent the first record of the species from the Iberian Peninsula. In addition, our molecular results strongly support that *Amphiduros pacificus* Hartman, 1961 from California (currently synonymised with *A. fuscescens)* requires to be re-described and reinstalled as a valid species. In turn, our morphological observations support suggesting all other non-Mediterranean reports of *A. fuscescens*, including the species still under synonymy (i.e. *Amphidrornus izukai* Hessle, 1925 and *Amphidromus setosus* Hessle, 1925) as likely being a cryptic species complex whose the taxonomic status requires further assessment.

## Introduction

*Amphiduros fuscescens* (Marenzeller, 1975) (Annelida: Hesionidae: Amphidurine) appears to be well-characterised by the absence of proboscideal papillae and emerging acicular chaetae, as well as by the presence of three antennae, eight pairs of tentacular cirri and inflated dorsal cirri with characteristic alternating length pattern and *in vivo* colouring (transparent, with median orange band and white spots and white tips). The species was described from the Italian coasts of the Adriatic Sea by Marenzeller (1875) and later reported from European waters in the Italian coasts of the Tyrrhenian Sea and the mainland French coasts of the Mediterranean Sea (Pleijel 1993, Pleijel 2001) and re-described, based on a syntype, newly-collected topotypes and additional Mediterranean materials (Pleijel 2001).

*Amphiduros fuscescens* is also known to occur in the North Atlantic Ocean in Brittany (Fage and Legendre 1927) and the Canary Islands (Núñez et al. 1997), as well as in the Red Sea (Israelian coasts of the Gulf of Aqaba), the Bismark Sea (Madang, Papua New Guinea), the Coral Sea (Great Barrier Reef, Capricorn Group, Australia) and the North Pacific Ocean (east Honshu and Kagoshima, Japan and from the US west coast to British Columbia) (Pleijel 1993, Pleijel 2001). This wide geographical distribution initially included several populations directly attributed to *A. fuscescens*, together with three species that were placed under synonymy by Pleijel (2001): *Amphidrornus izukai* Hesse 1925, *Amphidromus setosus* Hesse 1925 and *Amphiduros pacificus* Hartman 1961. Based on morphology (including descriptive and phylogenetic analysis), the available information did not allow Pleijel (2001) to validate the existence of more than one species and it was postulated that this situation (and we quote) ‘goes against common trends in both polychaete and other taxonomies, where species taxa tend to be split, and "cosmopolitan species" are regarded as artifacts which generally disappear on closer inspection’. We certainly agree with this statement, which has been confirmed by the most recent studies on polychaetes, particularly when molecular analyses are included (Hutchings and Kupriyanova 2018).

Our finding of *A. fuscescens* in the shallow waters of the Catalan Sea (Iberian Peninsula, NW Mediterranean) allowed us to describe some previously unreported morphological features (particularly based on living sexually mature specimens), as well as to discuss the taxonomic status and biogeographical distribution of the species and to contribute to the pool of known molecular information on Mediterranean marine fauna.

## Materials and methods

The specimens were collected in April 2019 and January 2021 in the Catalan Sea (NW Mediterranean) by turning up submerged boulders using SCUBA diving. One male and one female were found at 6-7 m depth in Punta Santa Anna, Blanes Bay and another female at 5 m depth at Cala Maset, Sant Feliu de Guixols (25 km northeast of Blanes). The worms were detached from the lower side of the boulders with the finger and immediately introduced in a plastic jar with native seawater. In the laboratory, the specimens were placed in a Petri dish with native seawater to observe them in living conditions. After adding drops of a saturated seawater/Thymol solution until the worms were relaxed, we obtained light microscopy photos of the specimens from Blanes with a CMEX 5 digital camera connected to a ZEISS Stemi CS–2000–C stereomicroscope. The female from Sant Feliu was not relaxed before being photographed with a Nikon D7200 digital camera equipped with a 105 mm macro-objective and two Sea&Sea YS-250 flashes.

Body and gamete measurements were made on the captured digital images using the measuring tools of the Adobe Photoshop CC version 2015.5, 1990-2016 Adobe Systems Incorporated. The male and the female from Sant Feliu were directly preserved in 96% ethanol for molecular analyses. The female from Blanes was fixed in a 4% formalin/seawater solution and then preserved in 70% ethanol for further morphological observations. Additional pictures of the preserved female were obtained with an SP100 KAF1400 digital camera connected to a Zeiss Axioplan compound microscope.

All specimens are deposited in the Museo Nacional de Ciencias Naturales of Madrid, Spain (MNCN).

### DNA extraction, amplification, sequencing and analysis

Total DNA was extracted from small pieces of the body wall of the specimens preserved in ethanol using DNAeasy Tissue Kit (Qiagen) and following the manufacturer’s protocol. A fragment (677 bp) of the mitochondrial cytochrome *c* oxidase subunit I (COI) was amplified using the primers ACOIAF 5’ CWA ATC AYA AAG ATA TTG GAAC 3’ and COIEU-R 5’TCD GGR TGD CCA AAR AAT CA 3’ (Zanol et al. 2010). PCR reactions were performed in a 25 µl total reaction volume with 0.15 µl BioTaq DNA Polymerase (5 U/ µl) (Bioline), 2 µl DNA template, 2.5 µl reaction buffer, 2 µl MgCl_2_, 1 µl Bovine serum albumin, 2 µl dNTPs (10 µM), 1 µl each primer (10 µM) and 13.35 µl milliQ water. The PCR temperature profile was as follow: 94 °C for 3 min, 35 cycles * (94 °C for 60 sec + 53 °C for 60 sec + 72 °C for 2 min) and 72 °C for 7 min. Electrophoretic gels were used to visualise PCR products to confirm fragment amplification. Successful amplifications were purified using ExoSAP-IT Express (USB) and sequenced in both directions (forward and reverse) by Macrogen, Inc. (Seoul, Korea). Overlapping sequence fragments were merged into consensus sequences using Geneious 8.1.8 (Kearse et al. 2012). Additional sequences belonging to other species of the Hesionidae Amphidurini tribe were obtained from GenBank (Table 1). COI sequences were translated into aminoacids, checked for stop codons in order to avoid pseudogenes and aligned together with GenBank additional sequences in Mesquite v.3.6 (Maddison and Maddison 2018). Sequences were compared with GenBank using the BLAST tool (Altschul et al. 1997A) and deposited to GenBank (Table 1). Genetic diversity was evaluated with uncorrected pairwise distances calculated with PAUP* v.4.0a161 and expressed as percentages (Swofford 2003).

## Taxon treatments

### Amphiduros fuscescens

(Marenzeller, 1975)

CC82D51B-BFDE-569C-8D43-2CC11EDFFA46

#### Materials

- **Type status:** Other material. **Occurrence:** recordedBy: Daniel Martin and Manel Bolivar; individualID: B214; sex: male; lifeStage: adult; preparations: relaxed with Thymol, fixed and preserved in ethanol 95%; associatedSequences: GenBank: MW135348; **Taxon:** scientificName: Amphiduros fuscescens; phylum: Annelida; class: Polychaeta; order: Phyllodocida; family: Hesionidae; **Location:** continent: Europe; waterBody: Mediterranean Sea; country: Spain; stateProvince: Catalunya; municipality: Blanes; locality: Punta Santa Anna; verbatimDepth: 6-7 m; verbatimLatitude: 41°40’26” N; verbatimLongitude: 2°48’07” E; verbatimCoordinateSystem: degrees minutes seconds; **Event:** samplingProtocol: Scuba diving; year: 2019; month: 4; day: 22; habitat: shallow sublittoral with boulders on mixed coarse sand and gravel; eventRemarks: the specimen was collected from the sea bottom, it was hidden below boulders; **Record Level:** institutionID: Museo Nacional de Ciencias Naturales; institutionCode: MVCN; collectionCode: MNCN 16.01/18935
- **Type status:** Other material. **Occurrence:** recordedBy: Daniel Martin and Manel Bolivar; individualID: XX214; sex: female; lifeStage: adult; preparations: relaxed with Thymol, fixed in a 4% formalin/seawater solution, preserved in 70% ethanol; **Taxon:** scientificName: Amphiduros fuscescens; phylum: Annelida; class: Polychaeta; order: Phyllodocida; family: Hesionidae; **Location:** continent: Europe; waterBody: Mediterranean Sea; country: Spain; stateProvince: Catalunya; municipality: Blanes; locality: Punta Santa Anna; verbatimDepth: 6-7 m; verbatimLatitude: 41°40’26” N; verbatimLongitude: 2°48’07” E; verbatimCoordinateSystem: degrees minutes seconds; **Event:** samplingProtocol: Scuba diving; year: 2019; month: 4; day: 22; habitat: shallow sublittoral with boulders on mixed coarse sand and gravel; eventRemarks: the specimen was collected from the sea bottom, it was hidden below boulders; **Record Level:** institutionID: Museo Nacional de Ciencias Naturales; institutionCode: MVCN; collectionCode: MNCN 16.01/18936
- **Type status:** Other material. **Occurrence:** recordedBy: Xavier Salvador Costa; individualID: X13; sex: female; lifeStage: adult; preparations: fixed and preserved in 95% ethanol; **Taxon:** scientificName: Amphiduros fuscescens; phylum: Annelida; class: Polychaeta; order: Phyllodocida; family: Hesionidae; **Location:** continent: Europe; waterBody: Mediterranean Sea; country: Spain; stateProvince: Catalunya; municipality: Sant Feliu de Guixols; locality: Cala Maset; verbatimDepth: 5 m; verbatimLatitude: 41°47’11” N; verbatimLongitude: 3°02’42” E; verbatimCoordinateSystem: degrees minutes seconds; **Event:** samplingProtocol: Scuba diving; year: 2021; month: 1; day: 30; habitat: shallow sublittoral with boulders on mixed coarse sand and gravel; **Record Level:** institutionID: Museo Nacional de Ciencias Naturales; institutionCode: MVCN; collectionCode: MNCN 16.01/18937

#### Description

##### Diagnosis

Gyptini with orange/brown eyes, dispersed eye pigment, coalescing nuchal organs, inflated dorsal cirri, and reduced stout emerging acicular notochaetae.

##### Morphological Description

**Figure 1.**
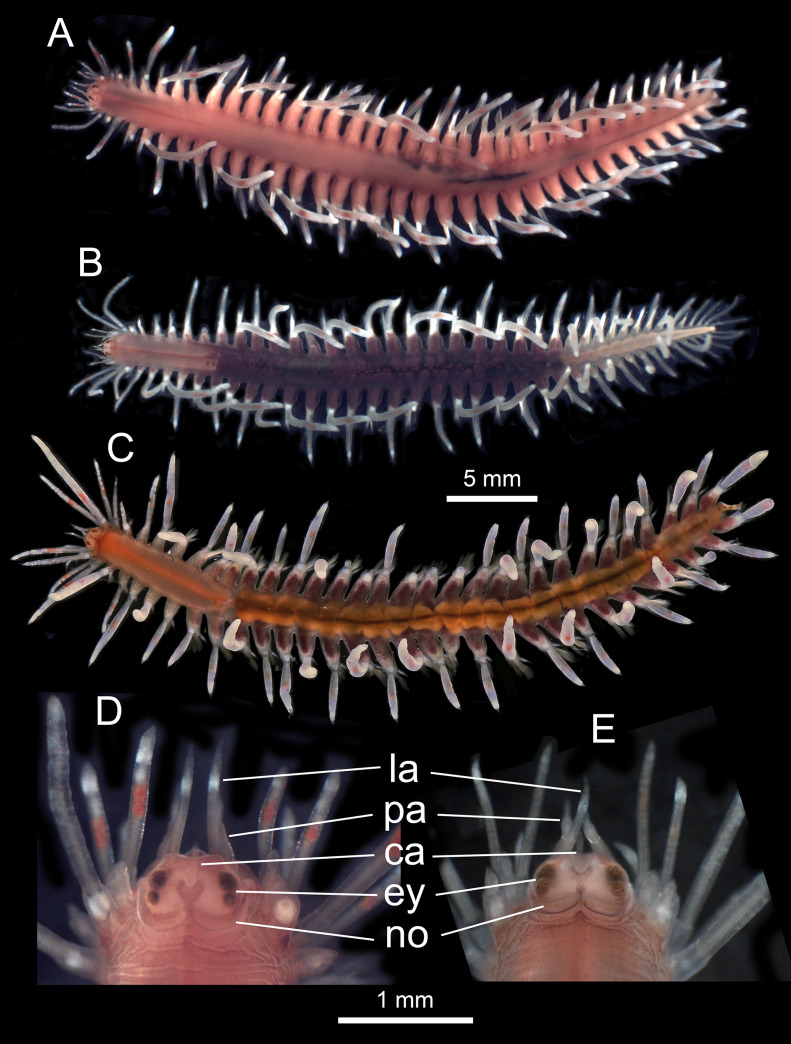
*Amphiduros fuscescens* (Marenzeller, 1857). Living specimens in dorsal view. **A–C.** Whole body; **D.** and **E.** Detail of the anterior end; A and D. Male from Blanes; B and E. Female from Blanes; C. Female from Sant Feliu; la: lateral antennae; pa: palps; ca: central antennae; ey: eyes; no; nuchal organs.

**Figure 2.**
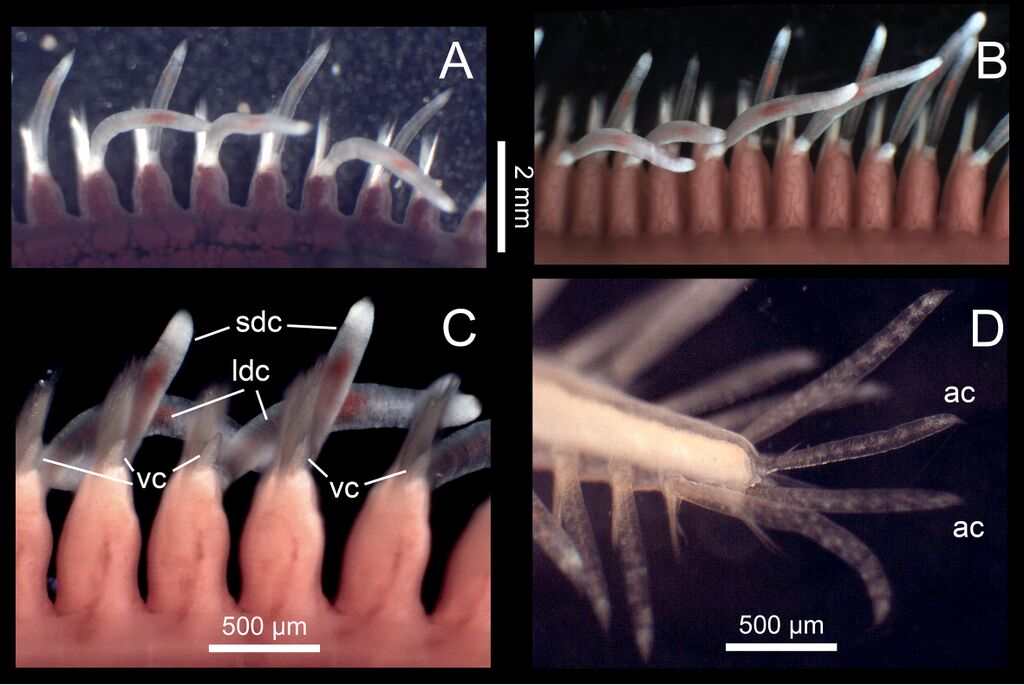
*Amphiduros fuscescens* (Marenzeller, 1857). Living specimens. **A.** Midbody parapodia of Blanes female, dorsal view **B.** Mid-body parapodia of Blanes male, dorsal view **C.** Mid-body parapodia of Blanes male, ventral view **D.** Pygidium of Blanes female. sdc: Short dorsal cirri; ldc: long dorsal cirri; vc: ventral cirri; ac: anal cirri.

**Figure 3.**
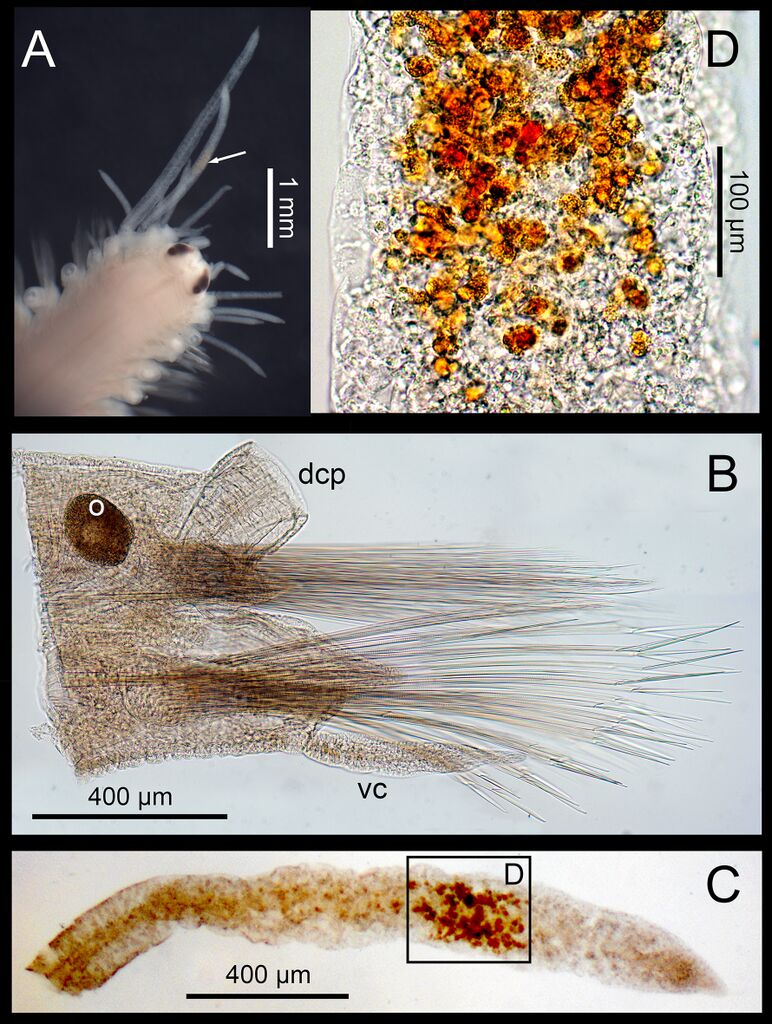
Blanes female of *Amphiduros fuscescens* (Marenzeller, 1857), preserved in formalin. **A.** Anterior end; white arrow pointing on the traces of the orange band; **B.** Mid-body parapodia; **C.** Short dorsal cirri; **D.** Detail of the orange band of the same short dorsal cirri. dcp: dorsal cirrophore; vc: ventral cirri.

Body anteriorly and posteriorly tapered, ventral flattened (Fig. 1A–C). Prostomiurn roughly oval, anteriorly truncate, posteriorly with distinct median incision (Fig. 1A–E). Palpophores cylindrical, antero-ventrally inserted and palpostyles proximally inflated, distally tapered (Fig. 1D–E). Lateral antennae longer than palps, cylindrical, distally tapering, inserted below palps; central antenna much shorter than lateral antennae, distinctly pointed, inserted dorsally between anterior pair of eyes, ceratophore absent (Fig. 1D–E). Anterior pair of eyes larger than posterior pair; nuchal organs long, bordering lateral and posterior margins of prostomium, mid-dorsally not coalescing (Fig. 1D–E). Dorsal cirri segment 1-5 elongated and slightly inflated, with those on segment 2, 4 and 5 largest and longest (Fig. 1A–C). Dorsal cirrophores segment 1-5 longer than on following segments. The remaining dorsal cirri roughly alternating inflated, with round tips, dorsally orientated and thinner, with pointed tips, more laterally orientated (Fig. 1A–C and Fig. 2A-C). In living specimens, thicker long dorsal cirri tending to be elevated from body plan, slightly sinuose, directed backwards; thinner short dorsal cirri not elevated from body plan, straight and perpendicular to antero-posterior body axis. Ventral cirri of segment 1-4 much longer than following ones, with those on segment 1 slightly longer; remaining ventral cirri ventro-distally inserted on neuropodium, with small and indistinct cirrophores and short, digitiform, distally tapering, non-pointed cirrostyles (Fig. 2C and Fig. 3A). Neuropodia and neurochaetae from segment 5, notopodia and notochaetae from segment 6. Notopodial acicular prechaetallobes conical, pointed; notoaciculae single or with small accessory basal acicula; notochaetae all chambered, simple and very fine, with numerous capillaries with two rows of small teeth and 2-10 most ventral curved chaetae with distal serration on ventral side; emerging acicular chaetae absent (Fig. 3B). Neuropodial acicular prechaetal lobes conical, much longer and more prominent than notopodial ones, neuroaciculae single or with small accessory basal acicula. Neurochaetae all compound, numerous, with chambered shafts and unidentate tips of blades; chaetal length increasing ventrally to dorsally (Fig. 3B). Pygidium with pair of large inflated cirri, similar to smallest dorsal cirri; median papilla present (Fig. 2D).

Colour in living animals transparent orange, with characteristically transparent appendages having white, iridescent bands on cirrophores and on tips of cirrostyles, a middle orange band on cirrostyles and white pigment as spots and bands on lateral antennae, dorsal cirri and cirrophores and on enlarged, anterior ventral cirri; eyes brownish; gut region orange (Fig. 1A–C and Fig. 2A–C). Preserved animals pale yellowish, eyes dark brown, other pigmentation lost, except for orange patches on cirrostyles of dorsal cirri when preserved directly with formalin (Fig. 3C–D).

##### Male sexually dimorphic characters

Body bright orange, 36.3 mm long, 5.7 mm wide (without parapodia) at chaetiger 15, with 35 chaetigers (Fig. 1A). Ratio body width vs. body length 0.4. Width of the long dorsal cirri 0.75 mm wide (0.1 when divided by body width). Posterior pair of eyes clearly separated from anterior pair (Fig. 1A and D). Sperm light orange, accumulating in parapodia from chaetigers 8–36. Most anterior (i.e. palps, antennae and fists, small tentacular cirri) and most posterior (i.e. last dorsal cirri and anal cirri) appendages brightly pigmented (Fig. 1A and Fig. 2B–C).

##### Females sexually dimorphic characters

Female from Blanes with violet body, 34.3 mm long, 3.8 mm wide (without parapodia) at chaetiger 15, with 40 chaetigers (Fig. 1B). Ratio body width vs. body length 0.11. Width of the long dorsal cirri 0.39 mm wide (0.08 when divided by body width). Posterior pair of eyes almost coalescent with anterior pair (Fig. 1B and E). Oocytes dark violet, from chaetigers 10–38, non-restricted to parapodia, measuring 115–175 µm in diameter. Most anterior (i.e. palps, antennae and fists, small tentacular cirri) and most posterior (i.e. last dorsal cirri and anal cirri) appendages brightly pigmented (Fig. 1B, Fig. 2A and D).

Female from Sant Feliu with orange/violet body, 37.8 mm long, showing traces of regenerating posterior segments, 5.3 mm wide (without parapodia) at chaetiger 15, with 40 chaetigers (Fig. 1B). Ratio body width vs. body length 0.14. Width of the long dorsal cirri 0.42 mm (0.08 when divided by body width). Posterior pair of eyes almost coalescent with anterior pair (Fig. 1C). Oocytes dark violet, from chaetigers 9–31, restricted to parapodia, measuring 90–115 µm in diameter. Most anterior (i.e. palps, antennae and fists, small tentacular cirri) and most posterior (i.e. last dorsal cirri and anal cirri) appendages brightly pigmented (Fig. 1B, Fig. 2A, and D).

#### Distribution

Southern France, eastern Sicily, northern Adriatic, north Iberian Mediterranean, Gulf of Aqaba. Other reports must be checked, as they may correspond to closely related, but distinct species (see Discussion).

#### Habitat

Below stones, amongst coarse shell gravel, shell and muddy sand and amongst kelp holdfasts, from shallow intertidal and medio-littoral. The species has been reported up to 50 m depth; however, these deeper reports may correspond to different species (see Discussion). Our specimens were observed to quickly swim by waving their bodies when the boulders below which they were hidden were turned up. Such a quick swimming reaction was always addressed prior to hiding again below close boulders.

## Analysis

### Molecular analyses

The Iberian male and female showed 99.8% and 100% similarity, respectively, to the COI sequences attributed in GenBank to *A. fuscescens* from SE French coasts (Ruta et al. 2007) (Table 1). The genetic divergence between the Iberian and French specimens ranged from 0.18% to 0.45%, while their genetic distance from *A. pacificus* exceeded 20% (Table 1).

### Taxonomic and morphological remarks

Some relevant morphological traits appeared to be linked to the reproductive status of the Iberian specimens. The two specimens from Blanes were collected in spring, on the same day, during the same dive and less than 5 m far from each other, while that from San Feliu was collected in winter, all of them having a similar length. However, the ratio of body width vs. body length was ca. 3 times higher in the male (0.4) than in the females (i.e. 0.11 in Blanes and 0.14 in Sant Feliu). Moreover, the male had the posterior pair of eyes clearly separated from the anterior pair, while the females had both pairs almost coalescent. As for the dorsal cirri arrangement characterising the species, both the male and the females kept it even after being relaxed with Thymol. The males had the long dorsal cirri 1.35-1.5 times wider than females; however, the widths of the cirri were comparable when divided by body width (i.e. 0.1 in the male vs. 0.08 in the females).

The male had appendages much more brightly pigmented than the females, as well as a brighter orange body. According to our observations, we suggest that this difference may be influenced by the evident presence of intra-coelomic gametes (i.e. light orange sperm and dark violet oocytes). This is supported by the difference in colour between the two females, which we assumed were in a different phase of the reproductive cycle depending on the collecting season. The female from Blanes (collected in spring) had larger oocytes not restricted to the parapodia and, thus, a darker body than the female from Sant Feliu (which was collected in winter and had smaller oocytes restricted to the parapodia).

Apart from the few characters linked to the sexual dimorphism mentioned above, the morphology of the Iberian specimens, both male and females, matched well with the re-description of the species by Pleijel (2001), based on specimens from SE France. Particularly, they coincided in their characteristic colour pattern. However, they slightly differed in eye colour that was brownish and dark brown, respectively in the *in vivo* and preserved Iberian specimens instead of orange and black, respectively in the live and preserved French specimens. The slight differences in colouring, here reported as an expression of sexual dimorphism, have never been reported in the previous descriptions of the species (Marenzeller 1875, Pleijel 2001). However, none of these previous authors indicated the reproductive status of the specimens they studied. Thus, we may assume that either they could be non-mature species or the slight differences were overlooked. Colouring quickly disappeared after fixation, particularly that on the body and parapodia (Fig. 2A and B), but also the white spots on cirri (Fig. 3C and D). This fading was almost immediate when using ethanol, while the worm preserved in formalin still kept traces of the orange pigments in the cirri, even several months after being transferred to 70% ethanol (Fig. 3A). This orange colouring was not evenly dispersed in the cirri tissues, but concentrated in irregularly round cells (10 to 20 µm in diameter, full of orange granules) (Fig. 3A, C and D). Fixation also affected the sexual products, as the sperm changed to a whitish colour and the oocytes became brownish (Fig. 3B).

The Iberian specimens also showed the characteristic alternating length and thickness of dorsal cirri as described by Pleijel (2001). However, this pattern was more specifically characterised by alternating thicker, sinuose cirri, elevated from body plan and backwards directed, with thinner, straight cirri, placed at the body plan level and perpendicular to the antero-posterior body axis. This particular arrangement of cirri in living specimens has not been previously reported. This particular disposition was observed both *in situ* and in the laboratory. Either it was maintained when the specimens were immobile or during their very fast waving displacements. Moreover, our specimens had the tips of the large inflated dorsal cirri round and those of the short dorsal cirri pointed, a peculiarity that was not mentioned by Pleijel (2001).

## Discussion

Our morphological observations clearly support the fact that the Iberian and French Mediterranean specimens belong to the same species, despite some slight differences in eye colouring, which could either be attributed to intraspecific variability, differences in reproductive status or preservation techniques. Thus, this will require to be confirmed by further observations. However, all other relevant morphological characters coincided. Therefore, taking into account that the French worms were used by Pleijel (2001) to re-describe the species, our morphological results clearly support the Iberian worms belonging to *A. fuscescens*. Despite the differences in the tips of the Iberian specimens (round in thick large cirri, pointed in narrow short cirri) and the existence of sexual dimorphism were not previously described, we strongly suggest that they probably also occur in the other Mediterranean populations. To date, the only known previous record of sexual dimorphism in Hesionidae was restricted to the specialised male copulatory organs in the interstitial genus *Hesionides* (Westheide 1979, Westheide 1967), so that the case, here described, is the second record for the family.

Based on COI sequences, the genetic distance between the male from Blanes, the female from Sant Feliu and the French specimens was lower than 0.5%. Therefore, our molecular data confirm the morphological observations and support all of them belonging to the same species. Moreover, our data also agree with those in Rouse et al. (2018) in supporting the differences between *Amphiduros* and *Amphiduropsis*, the latter being erected by Pleijel (2001), based on morphology. This author considered that the cosmopolitan distribution attributed to *A. fuscescens* cannot be contradicted, based on the morphological data available to date. This included different populations of the species all around the world, as well as those of *A. izukai*, *A. setosus* and *A pacificus* (all three under synonymy with *A. fuscescens*). However, we detected a 20% genetic distance between *A. fuscescens* (both the French and the Iberian materials) and *A. pacificus*. This was more than 10 times larger than the within-species threshold generally accepted as species discriminating in polychaetes (Meyer and Paulay 2005, Nygren 2014). This 20% was even higher than the distance between *Amphiduros* and *Amphiduropsis* (*Rouse et al. 2018*). These molecular data, together with the Californian origin of the specimens of *A. pacificus*, certainly support their belonging to a different species. Moreover, they also differ in adult size and colouring, being much larger and lacking the white pigmentation on the appendages typical of the Mediterranean *A. fuscescens*. Colour morphs in polychaetes have been consistently revealing to the existence of cryptic species-complexes (Nygren and Pleijel 2011, Aguado et al. 2019). Thus, we strongly support the taxonomic situation of *A. fuscescens* and *A. pacificus* merits being further clarified, with the latter at least requiring to be reinstalled, if not moved to a different, probably new genus.

Such a taxonomic clarification must be extended to all currently-known non-Mediterranean populations of *A. fuscescens* (i.e. from Canary Islands, Papua New Guinea and Australia), as well as to the other two synonymised species, *A. izukai* and *A. setosus* from Japan (Núñez et al. 1997, Pleijel 2001). Amongst them, there are evident differences in colouring, such as the blue pigmentation in the body and cirri of the specimens from Papua and Canary Islands. In particular, the Canarian specimens described by Núñez et al. (1997) also differed from the Iberian specimens in having orange eyes instead of brownish and from all Mediterranean worms in having the orange pigment more evenly distributed in the cirrostyles, instead of being concentrated in a middle band. Moreover, their long thick dorsal cirri were thicker (i.e. ca. 1.5 times) and their bodies were much shorter (i.e. 20 mm vs. 35 mm) than the Iberian specimens. Differences in body size also occurred in some of the other known populations, with the worms from Australia tending to be smaller and those from Japan being much larger than the Mediterranean ones (Núñez et al. 1997, Pleijel 2001). In addition, the dorsal cirri (both long and short) of living Japanese worms apparently lacked the white colour on tips and were much thicker than those of the Mediterranean specimens and the long dorsal cirri were more markedly club-shaped (http://miaw.o.oo7.jp/photo/Miura/Amphiduros.htm).

Therefore, we strongly suggest the existence of an unresolved complex of pseudo-cryptic species (*sensu* Nygren 2014) hidden amongst non-Mediterranean populations of *A. fuscescens*, comprising those of the three species currently under synonymy. Accordingly, *Amphiduros*, which is currently monotypic, would require further analyses, including morphological and molecular characterisation of the different populations. This would allow us to properly define the different morphotypes and to delimit the involved species. More likely, this would imply formal descriptions or re-descriptions either to erect new species or to reinstall some of the currently synonymised ones. Such a review must also take into account the possible existence of sexual dimorphism which, as we are here describing, based on the Iberian specimens, is revealed by slight differences in mature specimens, including colour, eye position and size and body and appendage proportions. However, despite the intrinsic interest of such a taxonomic revision, it certainly exceeds the objectives of the present study.

In summary, based on the specimens collected in the Catalan Sea, we report, for the first time, the existence of sexual dimorphism in a non-interstitial species of Hesionidae and the presence of *A. fuscescens* in the coasts of the Iberian Peninsula. Moreover, we describe some previously-undescribed morphological features in *A. fuscescens*, particularly the position of dorsal cirri in living specimens, while providing its westernmost Mediterranean report. Finally, we suggest the existence of a complex of pseudo-cryptic species involving the non-Mediterranean populations of *A. fuscescens*, highlighting that particularly *A. pacificus* requires to be further re-described and, more likely, reinstalled.

## Supplementary Material

XML Treatment for Amphiduros fuscescens

## Figures and Tables

**Table 1.** COI pairwise uncorrected–p distances (expressed as %) between the specimens of *A. fuscescens* of this study and the sequences previously published in GenBank.

	Accession Number	Origin	*Amphiduros fuscescens*			*Amphiduros pacificus*	Amphiduropsis cf. axialensis
			Blanes male	Sant Feliu female	Banyuls		
*Amphiduros fuscescens*	MW135348	Blanes, male	-				
	MW741554	Sant Feliu, female	0.30	-			
	DQ442561	Banyuls	0.18	0	-		
*Amphiduros pacificus*	JN631312	California	20.47	20.48	20.83	-	
Amphiduropsis cf. axialensis	MG640338; MG517506	Oregon; Costa Rica	17.55	17.66	17.07	18.95	-
*Gyptis mediterranea*	DQ442563	France	19.93	20.11	20.11	20.84	22.63
